# Errors of Diet

**Published:** 1905-03-15

**Authors:** Robert A. Gunn


### ERRORS OF DIET.

BY ROBERT A. GUNN, M.D.

Many fallacies regarding foods and their relative values in health and disease are still entertained by the public, as well as by many of the medical profession. In calling attention to some of these I trust I may be pardoned should I reiterate something I may have said in previous articles, or repeat what has long been known to the older members of the profession as established·truthh.

I have before said that, as a rule, people eat too much. I now add that excessive eating is almost iavariably the cause of the various disorders of the stomach and bowels, so frequently met with, especially among the denizens of large cities. Following these we have developed the various disorders of nutrition, as scrofula, rickets, rheumatism, gout, scurvy, diabetes and obesity.

When the stomach is overloaded, even with the best kinds of food, acid fermentation takes the place of normal digestion, and assimilation is arrested. Before the fermenting mass has time to pass from the stomach, another hearty meal is partaken of, and in a short time the stomach and bowels are so distended with gases as to render all the stages of digestion impossible. This is especially true of professional and business men, who lead sedentary lives, and whose brains are constantly active.

A case in point will illustrate this, and it is only one of thousands that come daily under the notice of physicians.

A prosperous broker, aged thirty-four, residing uptown, and doing business in Wall Street, New York, consulted me in February, 1892. When he reached my office he was gasping for breath, and it was half an hour before he recovered sufficiently to give me a history of his case. His skin was cold and clammy, his face, lips and ears were almost bloodless, his whole body trembled violently, and his countenance expressed fear as well as intense suffering. When he could speak he said he was suffering from heart disease, and that he had been treated by several physicians without obtaining relief.

On examination I found his entire abdomen enormously distended, his liver was enlarged and hardened, his heart palpitated violently, slight pressure over the stomach caused severe pain, and he seemed on the verge of collapse. His tongue was heavily coated with a thick brown coating, his bowels were constipated, and he constantly suffered from colic and flatulence. He said that he could sleep only in the semi-recumbent posture.

On inquiry I learned that for years he had eaten very hearty breakfast about eight o'clock, and immediately afterward took a car to his office. There his mind was actively engaged with the exciting events of a broker's life till one o'clock, when he went to lunch. He again ate heartily, and in ten or fifteen minutes he returned to his office, and continued his work till five or six o'clock. On reaching home he would eat a dinner of six or seven courses, and then start to his club or some place of amusement, and finish the day with a late supper.

When he finished his recital, I wondered that he had not broken down long before, as this had been his routine life for years.

He first began to suffer from the usual symptoms of dyspepsia, which were soon followed by sleeplessness, general nervousness, constant palpitation of the heart, marked emaciation, and finally a constant dread of sudden death from heart disease.

I first assured him that he had no organic disease of the heart, and that his entire trouble was with his digestive organs. I ordered him to drink two goblets of hot water every morning, and several times during the day, and to abstain entirely from eating as long as possible. I also prescribed a compound podophyllin pill every night, to act on the liver and move the bowels, and directed that he remain away from business for a time.

He went five days without taking any food, and experienced no inconvenience or desire for food after the first day. During this time the bowels moved freely several times a day, and the circumference of the abdomen was reduced seven inches.

In the meantime he was free from the palpitation of the heart and the constant distress in the stomach, and was willing to admit he was greatly improved.

For the next few days his diet consisted of bovinine, raw eggs and milk, administered in small quantities every two hours. At the end of the second week he felt so much better that he wanted to return to business. This I agreed to, with the understanding that he was to follow my directions implicitly. I insisted that he should only eat two meals a day—breakfast at 8:00 A. M., and dinner at 6:30 P. M. For breakfast he had chopped beef, broiled rare, with gluten bread and hot milk, and for dinner some plain animal broth with roast beef or mutton and gluten bread. He soon, however, varied his diet by adding eggs, bacon, fowl, fish, and green vegetables, still avoiding potatoes, rice, corn, white bread, pastries, puddings, and sweets of all kinds.

I also insisted that he should remain quiet half an hour after breakfast, and that then he should walk at least a mile on the way to his office. In the evening I ordered a rest of an hour before and an hour after dinner, while lunch and late suppers were strictly prohibited.

I also gave him a number of exercises to bring the muscles of the arms, chest and abdomen into action, which he practiced before retiring and on getting up in the morning.

In a short time he was entirely well, and has remained so until this day, and has increased in weight from one hundred and thirty to one hundred and seventy pounds.

I have seen hundreds of such cases where this plan has effected a cure. Try it, doctor, and leave medicines alone and I am sure you will be more than satisfied. With only two meals a day, the morning meal is entirely digested and removed from the stomach, which is thus left in a proper condition to receive and digest the evening meal.

About fifteen years ago, I, myself, began to have trouble with my stomach, especially after eating a hurried lunch. I discontinued my noonday meal, and have only eaten two meals a day ever since, and I have never had any further trouble, and have continued in perfect health.

Many persons find it impossible to eat certain kinds of food without a feeling of disgust or some unpleasant effect being produced. In such cases it is wrong to urge such foods on old or young, sick or well, on the supposition that they are healthful, as each person is better able to judge for himself what foods agree with him than any one else can judge for him.

Of course, a perverted appetite that craves for unnutritious and indigestible articles of food should not be gratified, for, in such cases, there is some abnormal condition that should be discovered and corrected.

As a rule, physicians pay too little attention to dietetics, and depend too much upon drugs. People should be made to understand the importance of thoroughly masticating their food, as the first step toward perfect digestion. No one can wash down their food with frequent swallows of fluids without sooner or later suffering from indigestion. No fluid should be taken during a meal, but each mouthful should be sufficiently masticated to cause ensalivation enough to render swallowing easy. After the meal a moderate amount of fluid may be taken, if desired, but not so much as to dilute the gastric juice, and thus retard stomach digestion.

The practice now so common, especially in large cities, of taking some strong stimulant before a meal to stimulate the appetite, and following this by an equally strong alcoholic cordial to aid digestion, is productive of much harm. These strong alcoholic beverages, taken at meal times, precipitate the pepsin of the gastric juice, and thus digestion is retarded. When taken after a meal they irritate the mucous membrane of the stomach, and cause a muscular contraction, which forces the food into the intestine before it is digested. Thus all benefit of the digestion of the proteid is lost, and the intestines have extra work to perform in throwing off the undigested mass as feces.

Persons advanced in years, or those whose digestive organs have been weakened by disease, may use some weak wine or beer with advantage, either before or after the meal, but in no case should the percentage of alcohol in these beverages be greater than eight or ten percent.

When stimulants are required, they should be taken midway between meals, and should always be largely diluted with water. When pure whisky or brandy is taken, it irritates the mucous surfaces of the stomach, and if persisted in it causes a congestion which soon destroys all normal function.

While alcohol has a place, both as a food and a medicine, its continuous use is productive of much harm, long before it leads to intoxication. I believe that it does harm, instead of good, when taken regularly by persons under twenty-five years of age, but for persons over fifty or fifty-five, when properly used it is often of great benefit.—*Medical Brief.*

